# Doctor-patient communication in the e-health era

**DOI:** 10.1186/2045-4015-1-33

**Published:** 2012-08-28

**Authors:** Jonathan P Weiner

**Affiliations:** 1 Department of Health Policy & Management, Division of Health Informatics, Center for Population Health Information Technology (CPHIT), Johns Hopkins University, 624 N. Broadway, Room 605, Baltimore, Maryland, 21205-1901, USA

**Keywords:** E-health, Health informatics, Electronic health records, Physician-patient communication, Israel, E-mail, Telephone, Health information technology, Information and communication technology, Mobile health, Health care policy

## Abstract

The digital revolution will have a profound impact on how physicians and health care delivery organizations interact with patients and the community at-large. Over the coming decades, face-to-face patient/doctor contacts will become less common and exchanges between consumers and providers will increasingly be mediated by electronic devices.

In highly developed health care systems like those in Israel, the United States, and Europe, most aspects of the health care and consumer health experience are becoming supported by a wide array of technology such as electronic and personal health records (EHRs and PHRs), biometric & telemedicine devices, and consumer-focused wireless and wired Internet applications.

In an article in this issue, Peleg and Nazarenko report on a survey they fielded within Israel's largest integrated delivery system regarding patient views on the use of electronic communication with their doctors via direct-access mobile phones and e-mail. A previous complementary paper describes the parallel perspectives of the physician staff at the same organization. These two surveys offer useful insights to clinicians, managers, researchers, and policymakers on how best to integrate e-mail and direct-to-doctor mobile phones into their practice settings. These papers, along with several other recent Israeli studies on e-health, also provide an opportunity to step back and take stock of the dramatic impact that information & communication technology (ICT) and health information technology (HIT) will have on clinician/patient communication moving forward.

The main goals of this commentary are to describe the scope of this issue and to offer a framework for understanding the potential impact that e-health tools will have on provider/patient communication. It will be essential that clinicians, managers, policymakers, and researchers gain an increased understanding of this trend so that health care systems around the globe can adapt, adopt, and embrace these rapidly evolving digital technologies.

## The e-health revolution is upon us

Communication and information technology is changing rapidly worldwide. This digital revolution will have a profound impact on how physicians and health care delivery organizations interact with patients and populations. Over the coming decade, face-to-face patient/doctor contacts will become less common and exchanges between consumers and providers will increasingly be mediated by electronic devices.

In their article in the current issue of the Israeli Journal of Health Policy and Research [1], Peleg and Nazarenko report on a survey they fielded within Israel's largest integrated delivery system. This survey describes the perspectives of patients regarding the use of mobile phones and e-mail to communicate directly with their physicians.

The value of this newly published paper is extended by a complementary paper by Peleg, Avdalimov, and Freud [2] that describes the perspectives of the physician staff at the same study site (the clinic network of Clalit Health Services in Israel's southern region) on this same issue.

These two papers add to the growing literature on the topic of changing modes of physician/patient communication in the digital age; in this case the use of direct access mobile phones and e-mail as an adjunct or alternative to face-to-face patient/doctor visits or more traditional clinic telephone lines [3-7]. It is fitting that this type of research is being done in Israel, given the nation's position as a global leader in the adoption of mobile phones, Internet use, and electronic health records [8-10].

The findings of these two Israel-based surveys offer useful insights to clinicians, managers, researchers, and policymakers interested in how best to integrate e-mail and direct-to-doctor mobile phones into their practice settings. But more importantly, they also provide an opportunity to step back and take stock of the dramatic impact that information & communication technology (ICT) and health information technology (HIT) will have on clinician/patient communication moving forward. Describing the scope of this issue, its potential impact, and future implications are the main goals of this commentary.

In highly developed health care systems like those in Israel, the United States, Europe, and even quite a few lower resourced systems, most aspects of the health care and consumer health experience are becoming supported and mediated by a wide array of technology. These ICT and HIT technologies include: electronic and personal health records (EHRs and PHRs), biometric & telemedicine devices that help diagnose or treat disease on a remote basis, and consumer focused wireless and wired internet applications ("apps") that aid in acquiring health-related knowledge, offering health-related social support, or providing a vehicle for voice or text communication with providers. Often the term "e-health" (for electronic-health) is used to refer collectively to all of these digital domains within the health care sphere.

This rapidly evolving e-health support infrastructure will forever change the way providers and consumers interact. It is essential that clinicians, managers, policymakers, and researchers gain an increased understanding of how this transformation will likely take shape so health care systems of the future can adapt, adopt, and embrace these technologies [11-16].

## Understanding the digital practice milieu

**Figure 1:**
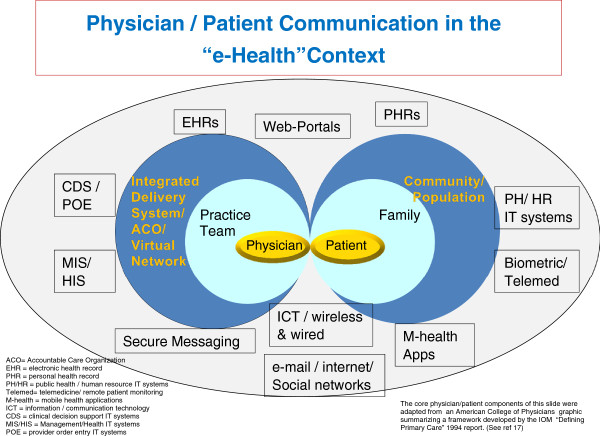
Physician/patient communication in the “e-health” context.

Figure 1 presents a graphic model of the digital practice milieu that will soon surround both the provider (aka the "supply" side) and the consumer (aka the "demand" side) of advanced health care systems like those in Israel, the United States, and other high income nations. This conceptual model acknowledges that in most modern health care systems the provider is no longer just a single physician or doctor group, but usually a multi-disciplinary team that is either physically or virtually integrated into an organized, structured delivery system (such as an Israeli sickness fund/health plan or an American integrated delivery system/accountable care organization). On the demand side, the model acknowledges that the "patient" (aka the consumer) is part of a family or other social network; something especially relevant where caregivers support young, old, or otherwise dependent patients. It is also critical to acknowledge that the family fits into a broader community, population, or societal context. This graphic gives emphasis to the special nature of the doctor/patient relationship as the figure builds upon a concentric ring model developed by the U.S. Institute of Medicine to help define an idealized role for primary care physicians [17].

Surrounding the provider and consumer concentric rings, Figure 1 lays out the many distinct (though often intertwined) types of e-health/HIT tools that currently, or in the near future, will likely be present to support and mediate information and communication flow in the health care systems of most developed nations.

Starting at bottom center of the chart (and front and center in the Peleg and Nazarenko paper) is the core of information/communication technology; telephone and broadband Internet networks. Specifically, this would include wired and wireless telephones and smart-phones and wireless and wired broadband. This is the platform on which a wide range of consumer-based Internet health applications are built, including social network support groups. It is also the platform for mobile phone (aka "m-health)-based apps that can be independent or "tethered" (i.e., linked) to a specific provider organization. ICT is also the backbone for conventional e-mail or secure messaging systems (which can be bi- or uni-directional between the provider and the consumer).

At the top center of the graphic model is the provider-controlled electronic health record, the consumer-controlled personal health record, and the so-called "web portal"; so termed because it is the web-based entry point for patients wishing to access their provider's EHR system. At its core, the EHR is the key repository and interactive source of medical information for the clinician and all other providers (and often the consumer). In advanced systems, the EHR serves as the hub for other key provider-based HIT components on the left side of the figure.

In evolved delivery systems the EHR goes beyond serving as just the "paperless" medical record. In such settings, the EHR serves as the core for most clinical and administrative processes through its linkage to the other provider-side HIT modules noted on the graph, including: clinical decision support (CDS) tools that help the doctor and other clinicians make evidence-based diagnosis and treatment decisions (e.g., tests needed to make a differential diagnosis, or how to choose the best drug); the "provider order entry" (POE) system that electronically implements clinical actions (e.g., e-prescribing, test ordering, or obtaining a specialist consult); and administrative/management information systems that support organizational and care management functions (e.g., patient outreach, quality improvement, patient scheduling, financial management or billing, and staffing).

Just as the EHR serves as the hub for clinicians, though not yet as widespread, the patient health record can serve as the hub for some of the consumer/community e-health functions summarized on the right side of the figure [18]. While this commentary and the featured paper focus on provider/patient interactions, it is important to remember that the locus where consumers manage most of their health needs is not within the provider organization. Rather, they address their health concerns within their family, workplace, school, and community settings. Therefore, thinking about health communication in this broader context is essential if the goal of individual and population wellbeing is to be achieved. Population/community centric delivery systems can help to achieve this goal by integrating their "medical care" e-health networks with home-based biometric/tele-medicine monitoring systems, personal health and wellness management m-health tools, and public and human services support systems. Integrating e-health solutions with this latter category of community-focused IT systems (often run by government agencies) is essential to address environmental, housing, food, and socio-economic needs and challenges. These "safety net" services must be part of the e-health equation, especially for those consumers at greatest risk.

## How e-health will impact doctor/patient communication

In a thoughtful recent essay on medical "professionalism" in the information age [19], David Blumenthal, a well-known American academic and former director of U.S. Federal HIT initiatives, identifies six ways in which the EHR and e-health tools described above will "enable and catalyze" changes within the profession of medicine. Following, using his six-part framework, I summarize and expand on some of his ideas with special reference to provider/consumer information flow and communication:

1) HIT and its embedded software will mediate almost all information and will be the source of almost everything that doctors and other clinicians will learn about their patients. As Blumenthal states, the "computer will be as omnipresent and important as the stethoscope"; [19]

2) Patient information will be accessible to all providers anywhere, anytime … 24/7. This access will be limited to only those clinicians to whom the patient grants overt access (e.g., by providing the password to their PHR) or tacitly (e.g., by accepting the default terms of EHR use within an integrated provider network);

3) Almost all patient/provider interactions will be mediated by the electronic HIT workflow (e.g., supported by digital guidelines and protocols) before, during, and after any clinician/patient contact. This will apply to physician/patient interactions that will be face-to-face, as well as those that are synchronous (i.e., "live") but not face-to-face, and those that are asynchronous (i.e., where the clinician and patient communicate directly, but at different times and places). Increasingly, before direct contact with the doctor can occur, electronic communication between the consumer and the delivery system will serve as an electronic triage process where in many cases consumers will get their needs met in other, usually digitally supported, manners not involving in-person encounters with the doctor;

4) Patients wishing to, can become full partners in their health care and wellness-enhancing processes. Such patients will have electronic access to almost as much information about their condition and the medical evidence base as their providers. This will include access (via web portals) to most of the information in their EHR and, increasingly, providers will "push" information to them electronically using e-health and m-health consumer tools;

5) The art and science of care surrounding the traditional face-to-face patient/doctor interaction will be forever changed as all aspects of communication, interaction, and information flow will become mediated (and monitored) by electronic tools [20]. How doctors and their delivery systems use these tools to diagnose, treat, and support the patient-centered needs of each individual, as well as the overall socially balanced needs of the community, will become a paramount goal of all high achieving clinicians and practice organizations worldwide. Perhaps ironically, this infusion of technology may make it possible, or even mandatory, that future clinicians focus more on the art of care given that the technical side of medicine will increasingly be handled by the IT "box". Physicians and other clinicians will be called on to serve as navigators and counselors to their patients who will potentially be faced with massive amounts of new information;

6) The HIT-mediated process will also dramatically change communication patterns between providers. HIT will enable all providers to work as a team and to coordinate their actions far more effectively even if they are not co-located. In the United States and other nations where large single site doctor/hospital integrated delivery systems do not (yet) dominate, the IT network will be the "digital glue" that holds together what are in effect virtual organizations [21].

## Learning from the Israeli e-health vanguard

As noted previously, there are few countries in the world with a digital infrastructure as prepared for e-health as Israel. Most consumers have Internet access and virtually all health care interactions in the nation are supported by a comprehensive EHR system. Moreover, most patient care is provided within several advanced, integrated private not-for profit delivery systems [22] that strive to increase quality and efficiency of care for both individuals and the enrolled population using HIT as a main driver. It is fitting to review a series of lessons relevant to doctor/patient digital communication that can be gleaned from the survey results of the featured study, as well as four other surveys and an observational study that focus on Israeli consumer and physician perceptions, behaviors, and knowledge in the domain of e-health.

The result of the Clalit southern region patient and doctor surveys [1,2], as well as two other recent surveys in the central region of Clalit, focusing on doctor/patient views on using e-health as a source of patient information [15,23], suggest that consumers and doctors in Israel (and probably elsewhere) have not quite figured out the rules of engagement for interacting with one another within the e-health milieu. Fully 88% of the patients believe having access to their doctor's personal cell phone number would improve the patient/doctor relationship and 71% of patients say likewise for access to their doctor's personal e-mail [1]. On the physician side at the same clinics, only 2% of doctors are currently willing to give out their direct dial cell phone numbers to all patients and only 3% are prepared to share their direct access e-mail with all patients [2]. However, if the organizational and reimbursement structure were redesigned, (e.g., if providers were paid for this time, or special clinic hours were earmarked for such communication), the great majority of physicians are open to fuller adoption of such new communication tools [2].

From the featured Peleg and Nazarneko article in this issue, it is encouraging that even though patients are interested in expanding the use of digital communication tools, they also have reasonable expectations. For example, most consumers are sensitive about not infringing on their doctor's private time and most agree that when a triage/call center is open, they should use this service before contacting their doctor directly [1].

In separate, but conceptually related studies, parallel surveys in the central region of the Clalit health organization asked doctors and patients about their views on consumer use of the Internet as a tool to support self diagnosis and management [15,23]. These surveys indicated that most Israeli patients in this region of Clalit avail themselves of such e-health tools (74%); but of the cohort that did so, only 19% of these e-health users share this fact with their doctor during their face-to-face interactions. Also, fully 78% of patients would like their doctors to provide guidance on how to use the Internet to help manage their conditions/problems [15].

The paired doctor survey in the central Clalit region provides an interesting set of mirrored responses, and unlike the e-mail/phone surveys in the southern region discussed above, doctors appear to be more ready to embrace the new technology than their patients. Even though most patients do not disclose to the doctor that they are making use of e-health information, over 80% of the primary care physicians said that when such information was brought into the exam room by the patient, they found this information to be satisfactory and indicative of the patient and/or their family being an appropriately activated consumer. It is also interesting that about 60% of doctors felt they needed further guidance on how best to incorporate patient use of e-health into their practice [23].

A recent paper by Neter and Brainan [24] reports the results of a national survey of over 4,200 Israelis, sponsored by the Israel National Institute for Health Policy and Health Services Research, the goal of which was to assess the degree to which the Israeli population is prepared for the new digital health care environment. Using an index measuring the achievement of "e-health literacy", this sophisticated analysis assesses how well various segments of the Israeli population are prepared for the new digital health milieu. Similar to studies in other nations, this survey identifies a "digital divide" between different segments of the society in terms of their ability to take advantage of e-health tools. One interesting finding is that those consumers who were more adept at using web-based health tools also scored significantly higher on independent measures of how well they interacted with their physicians. This is in agreement with the findings of the surveys at the Clalit central region, which suggested that doctors are primed to work with their "e-activated" patients as part of the health care interaction.

An interesting case study by Margalit et al. involving direct observations within the practices of three academic family physicians in northern Israel sought to assess the impact of the active use of EHR systems on doctor/patient communication within the face-to-face primary care encounter [25]. A detailed analysis of the visual and audio content of all doctor/patient interaction that occurred during a sample of face-to-face visits suggested that the computer had become a “third party" in the exam room. This was due in part to the workflow process that required the doctor to seek and input information using the EHR, thereby diminishing their time for effective inter-personal interaction with the patient. This study suggests that as technology continues to mediate between the clinician and consumer, human factors analysis and redesign of traditional care delivery processes will be paramount.

## Preparing for global health care “e-volution”

The digitalization process is well underway within many healthcare systems. But over the coming decades, in all reaches of the globe, clinicians, managers, policymakers, and scientists will need to work closely with consumers to plan, design, develop, implement, and evaluate the ever-expanding e-health infrastructure. There are many factors that must be in place for digital systems to be effective. Given that clinician/consumer communication and interaction is at the center of most e-health and HIT activities, before new technology can become fully ingrained within our health care systems, we must delineate, understand, and resolve many issues that surround this nexus. Some of the critical challenges and knowledge gaps that must be grappled with as these e-health systems become commonplace will include:

1) How e-health will impact traditional communication and interactions between clinical professionals, health care systems, consumers, caregivers, and communities.

2) How to design individual components of the ICT/HIT system to optimize provider/consumer communication and how best to educate and prepare all parties for effective communication within this new environment.

3) How best to integrate the many distinct "digital silos" representing the separate EHR, ICT, m-health, e-health, and IT components into a functionally integrated system that maximizes effective communication and interaction.

4) How to ensure that communication mediated by e-health systems is secure, confidential, and conforms to ethical principles.

5) How to shift from the past medical model of the 15 minute face-to-face, one clinician/one "patient" interaction, towards the concept of population health and wellness support in place 365 days a year, 24/7.

6) How to ensure that the disparities associated with a "digital divide" (i.e., between those with and without e-health tools and e-health literacy) can be surmounted in order to target the benefits of e-health to those with the greatest need (who currently may have the least access).

7) The literature on doctor-patient communication suggests that three key objectives of interaction during traditional (face-to-face) encounters are to create inter-personal relationships, exchange information, and to decide how best to treat the problem at hand [26]. In an e-health environment, how can the "inter-personal" human-to-human connection (which is all important to the healing relationship) be maintained, or even enhanced?

8) The design, development, and evaluation of e-health systems will require skills from multiple professional disciplines. How best should we pull together the required expertise which will include specialists with backgrounds in: inter-personal and mass communication, human factors, clinical sciences, health informatics and IT, computer science and engineering, public health/population sciences, and health management and policy?

9) How do we ensure that the design and implementation process is evidence-based? Given the significant cost of e-health systems and limited evidence to date regarding return-on-investment, it is essential that e-health effectiveness research become widespread. This line of work should be sure to incorporate a focus on the domain of provider/consumer interaction and communication. E-health tools are very amenable to the incorporation of fully integrated, ongoing e-supported performance assessment and monitoring, since most data items needed for the evaluation are already captured digitally and in real time [20].

10) Over the next several decades the "e-volution" of health IT, m-health, and other e-health systems will likely embody very rapid change and metamorphosis. In most cases this change will be disruptive to the current doctor/patient communication status quo. We will need to learn how to effectively manage the change and diffusion of e-health tools within professions, delivery systems, communities, and health care systems. For example, we must develop effective private and public e-health change management initiatives such as strategic planning, financial investment, research & development, market facilitation, training & education, evaluation, and regulation. As we plan, manage, assess, and expand e-health systems across nations and around the globe, we must never lose sight of the ultimate end game, which is to improve the health and wellbeing of the individual consumer and society-at-large.

## Competing interests

The author has no competing interests relevant to this commentary.

## Author information

At the Johns Hopkins University, Jonathan Weiner is professor of health policy and management and of health informatics, he also directs the newly established Center for Population Health Information Technology, as well as training programs in both health services research & policy and public health informatics. He is an internationally regarded health services researcher who has worked extensively in the primary care, managed care, quality measurement, case-mix, and e-health domains.

Commentary on the paper by Peleg, Nazarenko: Providing cell phone numbers and e-mail addresses to patients: The patient’s perspective, a cross sectional study. http://www.ijhpr.org/content/1/1/32/.
